# Alkaline phosphatase: Distinguishing between tuberculous and nontuberculous pleural effusion

**DOI:** 10.4103/0970-2113.53230

**Published:** 2009

**Authors:** Ashish Anantrao Jadhav, Jayashree Suhas Bardapurkar, Anuradha Jain

**Affiliations:** *Department of Biochemistry, People's College of Medical Sciences and Research Centre, By-Pass Road, Bhanpur, Bhopal, MP, India*; 1 *Department of Biochemistry, Government Medical College, Aurangabad - 431 001, Maharashtra, India*

**Keywords:** Alkaline phosphatase, differentiation, pleural effusion, tuberculosis

## Abstract

**Objectives:**

To evaluate the value of pleural fluid alkaline phosphatase and pleural fluid/serum alkaline phosphatase ratio for the purpose of differentiating tuberculous from nontuberculous pleural effusion.

**Materials and Methods:**

A total of 60 indoor patients, admitted to our hospital, having pleural effusion and suffering from varying etiologies, were included in this study. According to the final diagnosis, these 60 patients were divided into two groups: Tuberculous (30) and nontuberculous (30) pleural effusion.

**Results:**

The mean pleural alkaline phosphatase and pleural fluid/serum alkaline phosphatase ratio was significantly higher in tuberculous compared to nontuberculous pleural effusion. (*P* < 0.0001). In receiver operating characteristic curve analysis, sensitivity and specificity values were 90% and 80% for a cut-off value of 71 IU/L for pleural alkaline phosphatase activity; and were 90% and 86.66% for a cut-off value of 0.51 for pleural fluid/serum alkaline phosphatase ratio.

**Conclusion:**

From this study it is concluded that alkaline phosphatase activity remains a useful test in differentiation of tuberculous from nontuberculous pleural effusion.

## INTRODUCTION

Tuberculosis is still one of the important causes of exudative pleural effusion in our country.[1] Although, there are effective therapies for tuberculosis, epidemiological data shows a rise in incidence, especially since AIDS incidence rose steeply. The standard investigations to elucidate the etiology of pleural effusions are most often rewarding. However, sometimes investigations do not yield the exact etiologic cause of effusion, particularly in tuberculous pleural effusion.

Various parameters have been used to differentiate tuberculous from nontuberculous pleural effusion. These include: Pleural fluid adenosine deaminase,[2–4] PF lysozyme,[5] PF gamma interferon,[6,7] PF alpha 1-antitrypsin,[8] PF protease inhibitors,[8] PF CA 125,[7] PF tumor necrosis factor,[9] PF interleukin-1,[9] PF polymerase chain reaction,[6] and reported to be elevated in tuberculous pleural effusion.

Alkaline phosphatase (ALP) is one of the biochemical markers found in pleural effusion. Previous studies have used ALP either to differentiate between exudates and transudates[10–14] or to differentiate tuberculous from transudates of congestive heart failure.[15] This study was thus undertaken to confirm the usefulness of ALP activity in differentiating tuberculous from nontuberculous pleural effusion.

## MATERIALS AND METHODS

A total of 60 indoor patients, admitted to Government Medical College, Aurangabad, having pleural effusion and suffering from varying etiologies, were included in this study. In all cases, a standard clinical protocol was followed and routine laboratory tests of pleural fluid were carried out (total proteins, glucose, and pleural fluid cytology). Only the result of first thoracocentesis was used. Pleural fluid samples were cultured and pleural biopsy was also done to obtain a definitive diagnosis. According to the final clinical diagnosis achieved by standard methods, these 60 patients were divided into following categories:

1. A total of 30 patients with tuberculous pleural effusion in which diagnosis was confirmed when following conditions were met: Identifying bacillus in pleural fluid or biopsy specimen cultures. Presence of caseous granulomas in pleural biopsy tissue. Radiological and clinical evidence of tuberculous pleurisy. Response to antituberculous therapy.
2. A total of 8 patients with malignant pleural effusion in which diagnosis was confirmed by cytological examination of the fluid for malignant cells or by histopathology.
3. A total of 8 patients with parapneumonic pleural effusion in which diagnosis was confirmed when these patients have cough, fever and a radiographic pulmonary infiltrate that disappeared after antibiotic treatment.
4. Four patients of exudates other than the above causes, out of which one patient is of empyema having pus in the pleural cavity and a positive bacterial culture of pleural fluid; one patient of rheumatoid arthritis diagnosed clinically and as having rheumatoid nodule and increased serum rheumatoid factor; one patient of systemic lupus erythematosus diagnosed clinically and having LE cell phenomenon and antinuclear factor; one patient of liver abscess diagnosed clinically supported by aspiration of chocolate coloured pus from liver.
5. Ten patients of transudate, out of which three were of nephrotic syndrome diagnosed if the patient had proteinuria, edema and hypoalbuminemia; two patients of acute glomerulonephritis diagnosed if having oliguria, hematuria, hypertension, mild facial edema, granular casts in urine following a febrile illness; two patients of cirrhosis of liver diagnosed by liver biopsy in the presence of ascitis; one patient of congestive cardiac failure diagnosed due to cardiomegaly, radiological evidence of congested lungs, peripheral edema and response to treatment of congestive cardiac failure; one patient of severe hypoproteinemia having serum albumin level below 2 gm/dL; and one patient of renal failure diagnosed as there was raised urea and creatinine levels in the presence of clinical evidence of fluid overload.

Hence, the study was divided into two groups: Tuberculous (30) and nontuberculous pleural effusion (30) [Table 1]. Nontuberculous group consist of malignant effusion, parapneumonic effusion, empyema, rheumatoid arthritis, systemic lupus erythematosus, liver abscess, nephrotic syndrome, acute glomerulonephritis, cirrhosis of liver, congestive cardiac failure, severe hypoproteinemia, and chronic renal failure. Patients who met the diagnostic criteria of more than one of the previous categories or had pleural effusions of undetectable or obscure origin or had obvious hemothorax secondary to trauma, were excluded from study. Exudates were separated from transudates by pleural fluid/serum protein ratio greater than 0.5.[16]

**Table 1:** Causes of pleural effusion

Causes	No. of patients	% of patients
Tuberculous	30	50
Nontuberculous	30	50
Malignant effusion	08	13.33
Parapneumonic effusion	08	13.33
Other exudate	04	06.66
Transudate	10	16.66

The following studies were performed on the pleural fluid and serum of all patients: Pleural fluid ALP concentration (P ALP), serum ALP concentration (S ALP), pleural fluid/serum ALP ratio (P/S ALP ratio). Biochemical analysis of ALP was done on Erba Chem 5 plus semiautomatic analyzer using Erba Transasia Kits.

### Statistical analysis

Results were expressed as mean ± SD. Student's *t-*test was employed to determine statistical significance. *P* value less than 0.05 were considered statistically significant. Receiver operating characteristic (ROC) curves and areas under the ROC curves (AUC) with 95% confidence intervals were calculated for each of the criteria for evaluating the optimum cut-off points. In addition to using the cut-off points derived from ROC curves, the utility of each criteria for identifying tuberculous pleural effusion was evaluated by calculating the sensitivity, specificity, positive predictive value (PPV), negative predictive value (NPV) and efficiency.

## RESULTS

Out of 60 cases studied, 40 were men and 20 women. According to the clinical diagnosis, there were 30 cases of tuberculous pleural effusion of which 18 were men and 12 women with a mean age of 39.4 years (range 17-80). There were 30 cases of nontuberculous pleural effusion of which 22 were men and 08 women with a mean age of 38.2 years (range 03-72). In the group of patients with tuberculous pleural effusion, mean P ALP was significantly higher as compared to nontuberculous pleural effusion (*P* < 0.0001;) [Table 2]. Patients with tuberculous pleural effusion had a significantly (*P* < 0.0001;) [Table 2] higher mean P/S ALP ratio than with nontuberculous pleural effusion. However, such significant difference was not observed for S ALP levels. (*P* < 0.981;) [Table 2].

**Table 2:** Showing mean values (x) and SDs of ALP levels

	S ALP IU/L	P ALP IU/L	P/S ALP ratio
Tuberculous (n = 30)			
*x*	140.36	124.66	0.906
SD	43.21	58.69	0.370
Nontuberculous (n = 30)			
*x*	140.60	60.83	0.390
SD	32.80	56.51	0.280
*P* value	*P* < 0.981 (Nonsignificant)	*P* < 0.0001 (Significant)	*P* < 0.0001 (Significant)

P ALP level greater than 71 was observed in 27 out of 30 cases of tuberculous pleural effusion and six out of 30 cases of nontuberculous pleural effusion [Table 3]. P/S ALP ratio greater than 0.51 was observed in 27 out of 30 cases of tuberculous pleural effusion and four out of 30 cases of nontuberculous pleural effusion [Table 3].

**Table 3:** Cut-off points for ALP obtained by ROC curve analysis

Criteria	Cut – off point	Tuberculous (n)	Nontuberculous (n)
P ALP (IU/L)	≥71	27	6
	<71	3	24
P/S ALP ratio	≥0.51	27	4
	<0.51	3	26

n = number of cases

**Figure 1:**
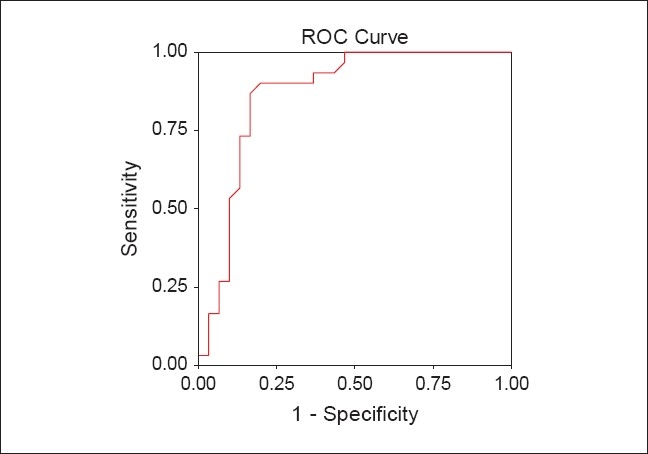
ROC plot of P ALP. Receiver-operating characteristic curve for P ALP levels, showing (1 - specificity) on the x-axis and sensitivity on the y-axis using different cut-off points of P ALP levels to arrive at the choice of the most appropriate cut-off point and which provided the greatest sum of sensitivity and specificity

**Figure 2:**
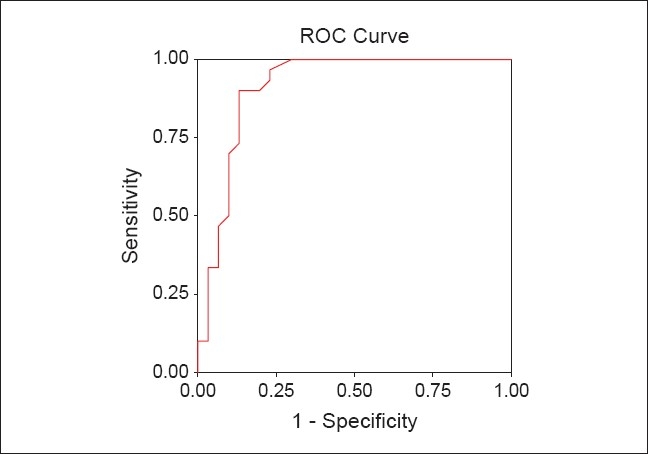
ROC plot of P/S ALP ratio. Receiver-operating characteristic curve for P/S ALP ratio, showing (1 - specificity) on the x-axis and sensitivity on the y-axis using different cut-off points of P/S ALP ratio to arrive at the choice of the most appropriate cut-off point and which provided the greatest sum of sensitivity and specificity

ROC plots of P ALP and P/S ALP ratio are shown in [Figures 1 and 2] respectively. The optimum cut-off level was determined by selecting points of test values that provided the greatest sum of sensitivity and specificity. The optimum cut-off levels for P ALP was 71 IU/L with sensitivity of 90.00% (95% CI 0.73-0.97) and specificity of 80.00% (95% CI 0.61-0.92). The area under the ROC curve was 0.865 [Table 4; Figure 1]. The optimum cut-off levels for P/S ALP ratio was 0.51 with sensitivity of 90.00% (95% CI 0.73-0.97) and specificity of 86.66% (95% CI 0.69-0.96). The area under the ROC curve was 0.911 [Table 4; Figure 2].

**Table 4:** Sensitivity, specificity, efficiency and AUC of ALP

Criteria	Sensitivity (%)	Specificity (%)	PPV (%)	NPV (%)	Efficiency (%)	AUC
P ALP	90.00	80.00	81.81	88.88	85.00	0.865
P/S ALP ratio	90.00	86.66	87.09	89.65	88.33	0.911

PPV-Positive predictive value; NPV-Negative predictive value

## DISCUSSION

The result of present study confirms that ALP activity is a useful parameter for differentiating tuberculous from nontuberculous pleural effusion. The value of P ALP and P/S ALP ratio were higher in patients with tuberculous pleural effusion as well as we found that the sensitivity and specificity of P ALP concentration to be 90.00% and 80.00% and for P/S ALP ratio 90.00% and 86.66% respectively for diagnosing tuberculous pleural effusion which was provided by ROC curve analysis [Table 4].

Alkaline phosphatase is a plasma membrane derived enzyme that hydrolyzes phosphate esters at pH 9. It is present in serum in six different forms, i.e., alpha 1-ALP, alpha 2–heat labile ALP, alpha 2–heat stable ALP, pre-beta ALP, gamma-ALP and leucocyte alkaline phosphatase. These different forms are due to the difference in the carbohydrate content (sialic acid residues). These activities arise from liver, bone, intestine and placenta.

Although, previous studies[12,14,17] have made an attempt to use ALP for differentiating tuberculous from other types of pleural effusion, but none of them have clearly differentiated tuberculous from nontuberculous pleural effusion. Francisco Carrion and Miguel Perpina[17] found in their study that P ALP was significantly raised in malignant pleural effusion as compared to tuberculous, nontuberculous and effusions due to miscellaneous causes. Further, while differentiating exudates from transudates, Muzaffer Metintas[12] found that P ALP and P/S ALP ratio were significantly raised in tuberculous pleural effusion as compared to neoplastic effusion, other exudates and transudates. However, Mushtaq A Lone[14] again while differentiating exudates from transudates reported that ALP did not differentiate tuberculous from other causes of effusion, including malignancy, parapneumonic effusion and nonspecific. In view of above controversy, we aimed to assess the value of ALP in differentiating tuberculous from nontuberculous pleural effusion and found that ALP is a useful biochemical marker for such differentiation.

To conclude, ALP is helpful in separating tuberculous from nontuberculous pleural effusion. However, further studies, involving larger number of patients, to evaluate the parameter covered in our study are needed in order to draw any conclusion or to achieve higher sensitivity.
